# Predictions of Genes Conferring Resistance to *Puccinia hordei* in an International Barley Panel Using Gene-for-Gene-Based Postulations and Linked Molecular Markers

**DOI:** 10.3390/plants14203150

**Published:** 2025-10-13

**Authors:** Davinder Singh, Laura A. Ziems, Karanjeet S. Sandhu, Mumta Chhetri, Miguel Sanchez-Garcia, Ahmed Amri, Mark Dieters, Robert F. Park

**Affiliations:** 1 Faculty of Science, School of Life and Environmental Sciences, The University of Sydney, Sydney, NSW 2570, Australia; laura.ziems@sydney.edu.au (L.A.Z.); karanjeet.sandhu@sydney.edu.au (K.S.S.); mumta.chhetri@sydney.edu.au (M.C.); robert.park@sydney.edu.au (R.F.P.); 2 International Centre for Agriculture Research in Dry Areas (ICARDA), Rabat 10170, Morocco; m.sanchez-garcia@cgiar.org (M.S.-G.); a.amri@cgiar.org (A.A.); 3 School of Agriculture and Food Sciences, The University of Queensland, Brisbane, QLD 4067, Australia; m.dieters@hotmail.com

**Keywords:** *Puccinia hordei*, barley, *Hordeum*, *Rph* genes, resistance, gene postulation

## Abstract

Deployment of resistant barley cultivars is the most cost-effective and environmentally responsible strategy to manage barley leaf rust caused by *Puccinia hordei*. Gene predictions based on screening of germplasm with an array of well-characterised pathotypes and application of molecular markers serve as a pivotal step for identification, characterisation, and deploying resistance in breeding programmes. We evaluated 77 barley genotypes from 17 countries using an array of diverse *P. hordei* pathotypes and molecular markers to predict resistance gene identities. Evaluation and resistance analysis of the panel determined four known all-stage resistance (ASR) genes—*Rph2*, *Rph3*, *Rph9.am*, and *Rph25* present individually or in combination, with *Rph3* being the most common (33% of entries) and *Rph2* the second most frequent (9%). Three entries, CG55, CG56, and CG57, exhibited low infection to all tested pathotypes and were negative for markers associated with *Rph7*, *Rph15*, and *Rph28*, potentially carrying novel uncharacterised resistance. In addition to ASR, our studies demonstrated that the core panel had a high prevalence of adult plant resistance (APR) to *P. hordei*, occurring in ~83% of entries. By employing markers linked to APR, we were able to partition known APR with *Rph24* found in the most lines (60%), followed by *Rph23* (17%), *Rph20* (14%), and uncharacterised (9%) either individually or in combination. The resistance sources identified in this study can be effectively utilised and combined by breeding programmes to diversify their resistance gene pool. Our study also revealed the virulence and avirulence profiles of 12 Australian *P. hordei* pts to catalogued *Rph* genes, providing pathologists and breeders with insights into combining genes relevant to their breeding regions and pathogen shifts.

## 1. Introduction

Barley leaf rust (BLR; caused by *Puccinia hordei*) continues to be a major challenge for barley production worldwide, affecting crops in various agroclimatic regions. Yield losses from *P. hordei* can be as high as 62% in susceptible varieties during epidemics [1], and reductions in quality such as shrivelled grain further reduce end-use value. Genetic resistance is considered to be the most cost-effective and environmentally sustainable management strategy to control BLR [2].

Resistance in barley to leaf rust can be broadly classified into all-stage resistance (ASR; expressed throughout the life stages of the plant) or adult plant resistance (APR; expressed only at adult plant growth stages). ASR is typically race-specific and governed by major gene/s that provide strong hypersensitive protection but often become vulnerable to breakdown due to evolving pathogen races as a consequence of migration, mutation, and recombination. In contrast, APR is usually race-non-specific, controlled by multiple minor genes, and provides partial but durable protection that becomes effective at post-seedling growth stages. The most effective and sustainable disease management approach involves combining both ASR and APR.

Gene postulation is a key tool for resistance identification and an important foundational step to identify, characterise and understand the genetic basis of resistance in germplasm collections and pre-breeding pipelines. This powerful well-established tool remains one of the most important to date for gene discovery. Gene postulation is based on Flor’s [3] gene-for-gene model. This model involves identifying resistance genes by analysing host responses to various pathogen races/pathotypes (pts) with distinct virulence or avirulence profiles. An infection type response array (ITRA) can be built by recording the phenotypic responses (high/compatible or low/incompatible) of a test line against multiple pts. By analysing ITRA and comparing phenotypic patterns to those of standard single gene differentials, the likely presence of one or more *Rph* (resistance to *P. hordei*) genes can be inferred. These postulations not only identify novel resistance genes in the test germplasm but also play a crucial role in mapping the global distribution of resistance, describing resistance in newly developed varieties, and identifying differential varieties for studying pathogen populations and their evolution [4,5]. The power of gene postulation has been enhanced by the increasing availability of molecular markers linked to resistance genes, especially those associated with APRs, most of which cannot be identified by multi-pathotype tests.

Germplasm collections are valuable repositories of genetic diversity that can be tapped to provide new resistance genes and desirable traits for crop improvement. The CAIGE (CIMMYT Australia ICARDA Germplasm Enhancement) programme is an Australian initiative that introduces, evaluates, and utilises high-yielding international barley germplasm to enhance local breeding capabilities [6]. The introduced lines undergo rigorous screening to identify traits such as yield potential, and resistance to abiotic stresses and major diseases. Over the past five years, CAIGE has introduced more than 2000 barley genotypes, and a preliminary evaluation has led to the establishment of a core panel for further detailed testing. We conducted intensive leaf rust testing of the CAIGE barley core panel to identify and characterise both ASR and APR to *P. hordei*. The study combined detailed greenhouse multi-pathotype seedling tests with adult plant field screening and molecular markers linked to known *Rph* genes. We also evaluated the virulence/avirulence of diverse Australian *P. hordei* pts against all known catalogued *Rph* genes to date.

## 2. Materials and Methods

### 2.1. Experimental Materials

**Figure 1:**
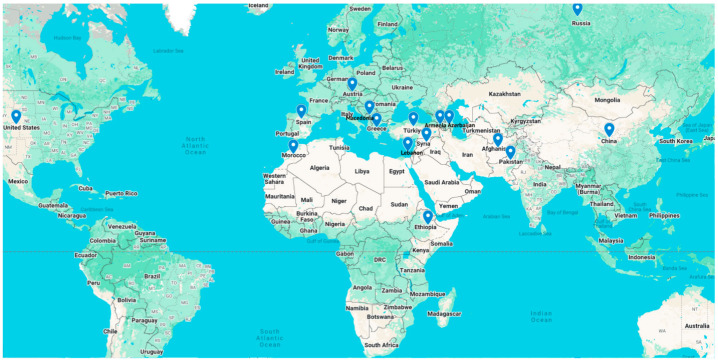
A world map showing the countries where the barley lines used in this study originated (for details of each line and origin, refer to Supplementary Table S1).

Resistance to *P. hordei* was investigated in a barley panel comprising 77 barley genotypes from 17 countries (Supplementary Table S1; Figure 1). The germplasm consisted of 39 breeding lines, 14 landraces, 11 single-plant selections, seven genetic stocks, one advanced cultivar, and five genotypes of unknown identity. The lines within the panel were targeted because they form part of the CAIGE barley core collection that is known to carry resistance to multiple diseases and to have high yield potential. A second panel of 30 barley genotypes comprising leaf rust differential genotypes and genetic stocks (Supplementary Table S2) was used to determine the virulence and avirulence profiles of the *P. hordei* pts used. The susceptible reference genotype ‘Gus’ was included in all tests. Positive controls for each gene were used for marker genotyping: Estate and Bowman + *Rph3* (*Rph3*), Cebada Capa and Bowman + *Rph7* (*Rph7*), PI333333 (*Rph15*), Flagship (*Rph20*), and Yerong (*Rph24*). The virulence profiles of 12 *P. hordei* pts ((*viz*. 200 P- (culture # 518); 220 P+ *Rph13+* (577); 222 P+ (545); 253 P+ (490); 4610 P+ (491); 5610 P+ (521); 5453 P- (560); 5457 P- (626); 5457 P+ (612); 5553 P+ (691); 5656 P+ (623); 5477 P- (672)) were assessed on 28 catalogued *Rph* genes carried by the respective differentials or gene stocks. The 77 test lines were evaluated with nine pathotypes (excluding 222 P+, 5453 P-, and 5457 P-) as they represent the same virulence profile and resolution as the 12 pts used for evaluating differentials and gene stocks. The pts were sourced from annual surveys of the pathogenicity of *P. hordei* in Australia and selected to represent pathogenic diversity within *P. hordei* populations in Australia [7,8].

### 2.2. Sowing Procedures and Plant Establishment

Greenhouse: Clumps of test lines, differentials and controls were sown and raised in 90 mm plastic pots, with 4 lines/pot (8–10 seeds/line). The potting media was formulated with a 4:1 ratio of composted pine bark and sand. Aquasol^®^, a soluble fertiliser manufactured by Hortico Pty Ltd. (Revesby, NSW, Australia), was then applied to the pots at a concentration of 25 g per 10 L. After sowing, the pots were moved to the seedling raising rooms, where they were kept at 22–24 °C until ready for inoculations (usually 10–12 days after sowing).

Field: The core panel and *Rph* gene stocks were assessed for APR to leaf rust at the Horse Unit (HRU) field site of the Plant Breeding Institute Cobbitty (PBIC), New South Wales, Australia (34°02′60.00″ S, 150°41′59.99″ E). The site receives an average annual precipitation over 800 mm. From 25 to 30 seeds of each line were sown in rows 60 cm long, with a space of 20 cm between rows in two replications. Gus was used as a disease spreader, sown after every five test lines to ensure uniform disease spread. Three to four weeks after sowing, the plots were fertilised with urea at a rate of 100 kg/ha and irrigated as necessary using fixed sprinklers. Weeds were either controlled with herbicides or removed manually when needed.

### 2.3. Inoculations, Disease Development, and Assessment

All stage resistance (ASR): Seedlings (approximately 10–12 days old) were inoculated with a suspension of *P. hordei* urediniospores (2 mg of urediniospores/1 mL of light mineral oil; Univar Solvent Naphtha L 100, Sydney, Australia). The inoculations were performed using an airbrush connected to a motorised compressor. Subsequently, the plants were transferred to an incubation chamber equipped with an ultrasonic humidifier to generate mist within the chamber. After the 24 h incubation period, the seedlings were relocated to microclimate rooms maintained at 22–24 °C with natural lighting and equipped with an automated irrigation system. Disease assessments were made approximately 10–12 days after inoculation using a modified infection type (IT) scale of ‘0’ to ‘4’, as described by [9]. ITs ‘0’, ‘1’ and ‘2’ were categorised as resistant, while ITs of ‘3’ or higher as susceptible. Variations in IT patterns were denoted by symbols: ‘-’: less than average for the class, ‘+’: more than average for the class, ‘c’: chlorosis, and ‘n’: necrosis. For example, 2 + c represents IT slightly higher than 2 but lower than 3, and it is associated with chlorosis (c). Similarly, 2-n IT is slightly lower than 2 but higher than 1, associated with necrosis. (n). If two different ITs are present on the same leaf and overlap, both types were recorded. For instance, 2 + 3 overlapping indicates the presence of both ITs 2 and 3 on the same leaf.

Adult plant resistance (APR): Field inoculations were carried as outlined in [10]. An Ultra-Low Volume Applicator (Microfit, Micron Sprayer Ltd., Bromyard, Herefordshire, UK) was used to spray a suspension containing urediniospores of pathotype 5457 P+ in Isopar oil at a concentration of 30 mg spores per 1.5 L of oil onto susceptible spreader ‘Gus’ on evenings when there was a high probability of overnight dew. Pathotype 5457 P+ was chosen because it is one of the most virulent and dominant pathotype in Australia, overcoming the maximum number of known ASR genes. Once the disease was established, disease score (DS) was recorded using a 1–9 scale, as described in [11]. Assessments were conducted at two distinct stages of crop development. The initial assessment was conducted approximately upon the emergence of the first spikelet, while the second assessment was conducted approximately upon the completion of anthesis, when the susceptible standard ‘Gus’ reached a susceptibility scale of 9. The two DS assessments were highly consistent, and second assessment was used for data analysis and generating graphical representations.

### 2.4. Marker Assisted Screening

DNA Extraction: Seedlings from all 77 lines and controls were grown in greenhouse, and leaf tissue samples from each line were collected from single plants of 10-day-old seedlings. Leaf tissues were subsequently dried using silica gel beads. DNA was extracted using the CTAB protocol as outlined by [12]. All samples were quantified using a spectrophotometer (Nanodrop™, Biolab, Melbourne, VIC, Australia) and DNA quality was assessed by electrophoresis on an 0.8% agarose gel. Diluted DNA (50 ng/µL) was used in all PCR reactions.

Genotyping: All lines were genotyped using markers: bPb-0837 (dominant) and sun690-691 (codominant), linked to *Rph20* [13,14], Ebmac0603, linked to *Rph23* [15], and sun43-44, linked to *Rph24* [14]. Lines postulated to carry *Rph3* were validated with marker MLOC_198 [16]. Lines resistant to all pts at seedling growth stages were additionally screened with markers Hvpg4, K3 and M5 linked to the ASR genes *Rph7* [17], *Rph15* [18], and *Rph28* [11], respectively. Table 1 provides detailed profiles and assays of all molecular markers used in this study.

### 2.5. Gene Postulations

The *Rph* genes were postulated by comparing the IT patterns of test entries with those of resistant controls. A high infection type (HIT) on a test line indicated the absence of a resistance gene for which the specific pathotype was avirulent. For instance, an *Rph1*-avirulent pathotype that produced a HIT on a given line indicated the absence of *Rph1* in the test line. The test lines exhibiting similar low IT (LIT) patterns to the control, suggested the presence of the same genes. If the IT pattern for the seedlings of a test line did not align with the IT pattern of the reference gene or showed a negative result for the marker, it was classified as an unidentified seedling resistance (UASR). The APR genes were determined based on the presence and absence of linked markers. Any line that lacked the *Rph20*, *Rph23*, and *Rph24* markers but still exhibited APR was postulated to carry an uncharacterised APR (UAPR). Where applicable, the pedigrees were also considered during the postulation process. APR carrying lines were classified in three categories based on levels of resistance: [high APR (HAPR), disease score (DS) 1–3.5], [moderate APR (MAPR), DS 3.6–5], and [low APR (LAPR), DS 5.1–7.5] Lines with disease score > 7.5 were considered susceptible.

## 3. Results

### 3.1. Virulence Diversity of Australian P. hordei Pathotypes

**Figure 2:**
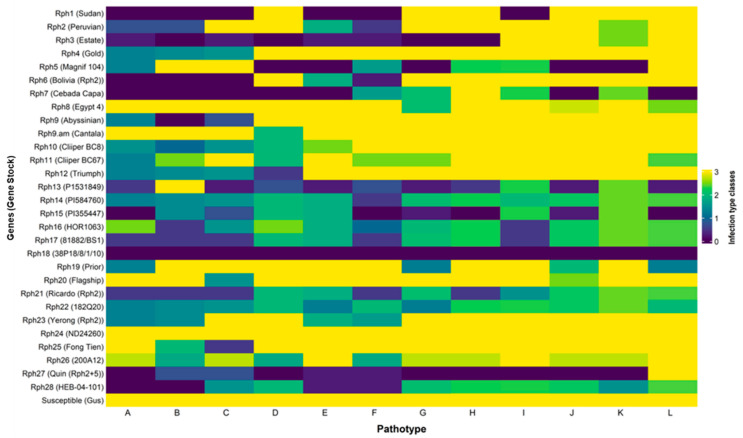
Virulence-Resistance interaction Heatmap: 12 pathotypes × 28 *Rph* genes. A = 200 P- (518); B = 220 P+ *Rph13*+ (577); C = 222 P+ (545); D = 253 P+ (490); E = 4610 P+ (491); F = 5610 P+ (521); G = 5453 P- (560); H = 5457 P- (626); I = 5457 P+ (612); J = 5553 P+ (691); K = 5656 P+ (623); L = 5477 P- (672).

Twelve Australian *P. hordei* pts, when tested on 28 catalogued *Rph* genes, exhibited diverse virulence spectra (Supplementary Table S2; Figure 2). Genetic stocks and differentials carrying *Rph1* to *Rph12* and *Rph19* displayed a range of low and high ITs, consistent with their designated formulae assigned by [8].

*Rph13* exhibited low ITs to all pts except *Rph13*-virulent pt 220 P+ *Rph13*+, while *Rph25* demonstrated ineffectiveness against all pts except 220 P+ *Rph13*+. Genes *Rph14* to *Rph18*, *Rph21*, *Rph22*, and *Rph28* all produced incompatible ITs to all test pts, although LITs among the *Rph* genes displayed a range, from hypersensitive to intermediate. Quinn, a carrier of *Rph27* was resistant to all pts except 5477 P-; however, the LIT of *Rph27* in Quinn corresponds solely to pathotype 222 P+ because Quinn possesses additional genes *Rph2* and *Rph5*, and 222 P+ exhibits virulence against *Rph2 + Rph5*. Pathotype 5477 P- is virulent on all three genes (*Rph2 + Rph5 + Rph27*) carried by Quinn. Pathotype 5553 P+ was the only pathotype virulent on *Rph7*. It was similar in virulence to pathotype 5453 P- but with additional virulence for *Rph7* and *Rph19*.

Genetic stocks of three APR genes, *Rph20*, *Rph23*, and *Rph24*, exhibited HITs to all of the pts in seedling tests, as anticipated given their APR gene status. The LIT observed in Yerong (*Rph23 + Rph2*) to four pts (220 P-, 220 P+ *Rph13+*, 4610 P+, and 5610 P+) is attributed to the presence of *Rph2* carried by Yerong.

**Figure 3:**
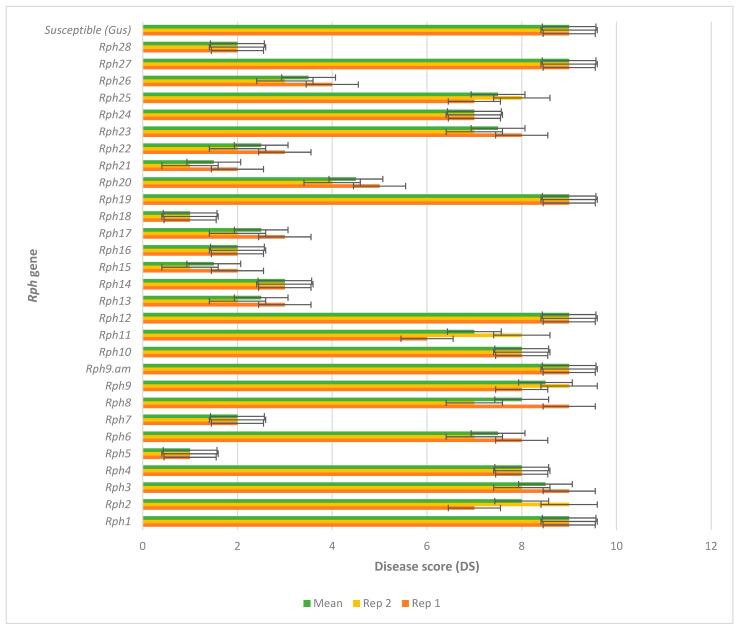
The mean (green bars) field disease score (DS) of 28 *Rph* genes over two replications (yellow and orange bars) with pathotype 5457 P+. DS was aggregated using a 1–9 scale for each replication and subsequently averaged for the two replications.

The 28 lines when tested as adult plants in the field over two replications using pt 5457 P+ produced variable disease responses. Eleven lines were classified as resistant (*Rph5*, *Rph7*, *Rph13*, *Rph14*, *Rph15*, *Rph16*, *Rph17*, *Rph18*, *Rph21*, *Rph22*, and *Rph28*), two as moderately resistant (*Rph20* and *Rph26*), five as moderately susceptible (*Rph6*, *Rph11*, *Rph23*, *Rph25*, and *Rph26*), and 11 as susceptible (*Rph1*, *Rph2*, *Rph3*, *Rph4*, *Rph8*, *Rph9*, *Rph9.am*, *Rph10*, *Rph12*, *Rph19*, and *Rph27*) (Figure 3).

### 3.2. Gene-for-Gene-Based Gene Postulations

A wide spectrum of ITs was observed on 77 test lines when tested with 9 pts (Supplementary Table S3). The generated ITs were compared to gene stocks carrying known *Rph* genes to predict IT-specific *Rph* genes involved. This approach identified both known and novel ASR genes (Table 2). Of the 77 lines tested, 22 exhibited seedling susceptibility (IT “33+” to “3+”) to all nine pts (Supplementary Table S3). Consequently, these lines lacked ASR genes that could be detected using the array of pts employed.

LITs were recorded for 26 lines (CG30–35, 36–42, 47–49, 51, 52, 58–61, 67, 68, 70, and 71), with all pts except *Rph3*-virulent pts 5457 P+, 5477 P-, and 5656 P+ (Table 2; Supplementary Table S3). This IT response was similar to that of the *Rph3*-carrying differential line Estate, leading to the postulation that all 26 lines carry *Rph3*. Eight lines (CG1, 13, 26, 62, 69, 72, and 77) were postulated to carry *Rph2* as these lines exhibited LITs to pts 200 P-, 220 P+, 4610 P+, and 4610 P+ (avirulent on *Rph2*) and high ITs to all other pts (virulent on *Rph2*), an IT pattern similar *Rph2*-carrying differential Peruvian (Table 2; Supplementary Tables S2 and S3). CG77 showed heterogeneity in its *Rph2* response.

*Rph25* was postulated in three lines (CG25, 45, and 73) because these lines exhibited HIT with all pts except 220 P+ *Rph13*+, a pattern that corresponds to *Rph25* and its donor, Fong Tien (Table 2; Supplementary Tables S2 and S3). Lines CG23 and CG76 were postulated to carry *Rph25* and *Rph9.am*, as these two lines showed incompatible responses to 253 P+ (the only pt avirulent on *Rph9.am*) and pt 220 P+ *Rph13*+. Lines CG6, 50, and 53 were also postulated to carry *Rph9.am*, but CG6 and 53 carried *Rph9.am* in conjunction with an unidentified resistance differing from *Rph25*, as these lines exhibited HIT with 220 P+ *Rph13*+ but low with 253 P+ and 200 P-. CG54 was the only line showing an IT response (high with pts 220 P+ *Rph13+* and 5477 P-, and low to all other seven pts) that matched *Rph5* carried by differential Magnif 104 (Table 2; Supplementary Tables S2 and S3), so it was postulated to carry *Rph5*.

Nine genotypes (CG2, 14, 24, 28, 29, 43, 64, 65, and 74) exhibited resistance phenotypes against at least one pathotype that did not match any genetic stock (Supplementary Table S3), indicating the presence of UASR in each. Three lines (CG55, 56, and 57) exhibited LITs to all test pts.

### 3.3. Field and Marker-Based Assessment of APR

**Figure 4:**
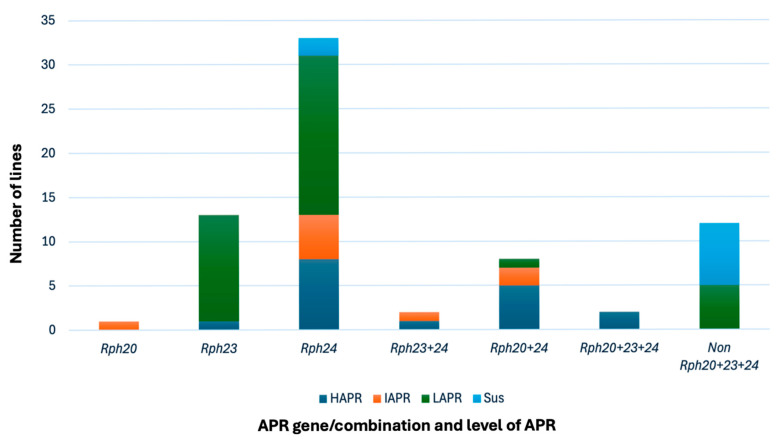
Frequency distribution and dissection of APR based on known APR genes *Rph20*, *Rph23* and *Rph24*, and levels of APR carried by each gene individually or in combination. *Rph20*, *Rph23* and *Rph24* determined based on linked molecular marker and APR levels determined as high (HAPR, DS = 1–3.5); moderate (MAPR, DS = 3.6–5); low (LAPR, DS = 5.1–7.5); susceptible (DS > 7.5) against pathotype 5457P+.

The 77 lines showed a range of disease severities when evaluated in two randomised replications. The data were highly correlated (r^2^ = 0.83) for the two replications, so the average of the two was used for further analysis of APR. Out of the 69 lines that were seedling susceptible to pt 5457 P+, 58 showed varied levels of resistance in field where the same pathotype was applied. These lines were postulated to carry APR and are classified into three categories based on resistance levels: 15 with HAPR, eight with MAPR, and 36 with LAPR (Table 3; Figure 4). Of the nine lines (CG28, 29, 34, 43, 54, 55, 56, 57, 64) resistant to pt 5457 P+ at the seedling stage, eight showed high levels of resistance and one showed low level under field conditions. However, the APR in these nine lines was undetermined due to the presence of effective ASR to 5457 P+ in each.

To ascertain whether the APR identified was due to one or more of the three catalogued APR genes (*Rph20*, *Rph23*, and *Rph24*) or whether it was novel, all APR-carrying lines were subjected to molecular marker screening using bPb-0837, sun690-691, Ebmac0603, and sun43-44, which are closely linked to *Rph20*, *Rph23*, and *Rph24*, respectively. Marker analysis revealed the presence of *Rph24* singly in 33 lines (CG1–4, 7–23, 25, 28, 30, 33, 46, 48, 49, 69, 71, 73, 74, and 77; Table 3; Figure 4). *Rph23* was carried by 13 lines (CG35–41, 50, 56, 59–61, and 67) singly and by two (CG24 and 71) in combination with *Rph24*. *Rph20* was postulated in 1 line (CG75) singly and 10 lines (CG26, 34, 43–45, 62, 64–66, and 72) in combination with *Rph24* based on dominant as well as codominant markers. Two lines (CG63 and 76) carried combinations of all three genes, *Rph20*, *Rph23*, and *Rph24* (Table 3).

## 4. Discussion

Gene postulations using an array of well characterised pathotypes is a highly efficient and cost-effective method for identifying characterised ASR genes and novel sources of rust resistance in cereals. The findings from such rust tests can be validated further through the application of linked molecular markers where available [19]. By employing a combination of these two methodologies, our studies successfully characterised four known ASR *Rph* genes (*Rph2*, *Rph3*, *Rph9.am*, and *Rph25*) either singly or in combination in a panel of high yielding international barley genotypes. The presence of these genes has also been extensively reported in other germplasm collections in research undertaken at the University of Sydney’s Plant Breeding Institute [2,20,21].

Of the identified genes, *Rph3* was the most commonly detected (present in 33% of the lines), followed by *Rph2* (9%). The presence of *Rph3* in these lines was further confirmed using a marker linked to *Rph3* [16]. Gene *Rph3* is common in global barley germplasm and has been used extensively in commercial barley cultivars worldwide [2]. The widespread exploitation of *Rph3* likely led to its enrichment in breeding programmes, and consequently, the presence of this gene in numerous modern commercial barley cultivars is likely a result of gene enrichment in breeding populations rather than intentional deployment. *Rph2* is a complex locus with multiple alleles, as evidenced by varying resistance levels conferred by numerous *Rph2* donors [1,22]. In this study, all *Rph2* postulated lines showed similar LITs, ranging from “;1cn” to “;12+cn”, except for CG1 and 62, which showed *Rph2* race specificity but produced ITs that were either elevated (“2c” to “2 + 3+c” for CG1) or reduced (“;+n” for CG62). Further allelic studies are recommended on these lines. The genotypes in which *Rph2* was detected in our study originated from five countries: Lebanon, Morocco, Russia, Syria, Turkey, and the USA, suggesting its global widespread distribution. The four known ASR *Rph* genes identified in this study possess limited utility in resistance breeding because virulence is readily available for all these genes to Australian *P. hordei* populations and globally [1,9].

Three lines (CG55, 56, and 57) in this study are of high importance as they demonstrated resistance to all *P. hordei* pts tested and may possess novel, uncharacterised resistance. To eliminate the possible presence of *Rph7* and *Rph15/Rph16* in these lines, we tested three lines with perfect markers linked to *Rph7* [17] and *Rph15/Rph16* [18] and *Rph28* [11]. All three lines lacked markers associated with the respective resistance, indicating the absence of *Rph7*, *Rph15*, and *Rph28*. Virulence for *Rph7* was recently detected in Australia (Park, RF unpublished), and testing with the *Rph7*-virulent pathotype 5553 P+ further ruled out the presence of *Rph7* singly in these lines, as all three were resistant to this pathotype. If *Rph7* is present in these lines, it must be in combination with another ASR for which pt 5553 P+ is avirulent (e.g., *Rph5*). *Rph17* and *Rph18* are also effective against all known Australian pts [1], but they are unlikely to be carried by these lines as both originate from *H. bulbosum* and the introgression lines carrying these resistances were not used in developing the germplasm tested. Based on this critical analysis, it is highly probable that CG56, CG57, and CG58 represent novel sources of ASR to *P. hordei*. All three lines originate from Morocco and share Canela//E.acacia/DEFRA pedigree of their female parent, potentially contributing to their resistance. Genetic analysis and allelic studies on these lines are recommended to determine the novelty of the resistance genes involved.

Many breeders favour using APR over ASR due to its reputation as a durable form of resistance [2]. To date, only three APRs have been designated for resistance to *P. hordei*: *Rph20* [13], *Rph23* [15], and *Rph24* [23]. In this study we investigated the architecture of APR in the core panel with an aim to characterise and pinpoint known APR, while also discovering new sources of this important resistance. Our study found that the CAIGE core panel had a high prevalence of APR to *P. hordei*, occurring in 64 out of 77 tested lines (~83%).

With the application of markers linked to APR, we were able to partition known APR with *Rph24* found in the most lines (~60%), followed by *Rph23* (~17%) and *Rph20* (~14%) either singly or in combination. The level of APR contributed by each gene and combinations varied among lines. Lines with a single gene (*Rph23* or *Rph24*) showed LAPR to MAPR. Lines with a two-gene combination showed MAPR or MAPR to HAPR, while those with all three genes showed HAPR. These findings corroborate previous research [2,23] that demonstrated that three known APR *Rph* genes *Rph20*, *Rph23* and *Rph24* in barley are interactive and additive, and provide optimal resistance when used in combination, particularly three gene combinations. Five lines in our study (CG 45, 52, 56, 61, and 73) showed APR that based on marker genotyping differed from the three known APR genes. The resistance carried by these lines was minor but comparable to *Rph23* and *Rph24* when these genes are present alone. While these unidentified minor APRs may not provide adequate protection individually, they could contribute to gene additivity when combined with the other three APRs, and hence further studies are recommended to understand their additivity. Eight more lines postulated to carry *Rph24* had high APR levels compared to typical *Rph24* moderate to low resistance levels, indicating they likely carry uncharacterised APRs in addition to *Rph24*.

This study not only characterised known and new resistances but also contributed to pathogen surveillance by linking host resistance patterns to virulence trends. The virulence profile of Australian pts is well understood [1,8], but it has not been documented as a single reference for all the 28 *Rph* genes that have been catalogued to date. Our study provides a comprehensive analysis of the virulence and avirulence profiles of 12 diverse Australian pathotypes of *P. hordei* using genetic stocks encompassing all known catalogued genes, thereby providing a comprehensive reference source for further research. The shifts in virulence and avirulence profiles of historic pts serve as predictive indicators of which resistance genes may be at risk and provide timely warnings for breeders to modify their resistance breeding strategies. The information generated also provides a guide as to whether or not there is a need for the refinement of differential sets employed in race pathotyping. This comprehensive approach enhances host and pathogen monitoring and facilitate long-term disease control by characterising resistance genes and their effective deployment.

In summary, these studies have identified and characterised known and novel sources of ASR and APR. Five lines (CG 29, 55, 63, 76, and 77) were identified to carry high levels of seedling and/or APR. These sources can be effectively utilised and combined by breeding programmes to diversify their resistance gene pool and reduce reliance on single major-effect genes, which are more vulnerable to breakdown. At the same time, this study reveals the virulence and avirulence profiles of Australian *P. hordei* pts, giving pathologists and breeders strategic insights into combining genes relevant to their breeding regions and pathogen shifts.

## Figures and Tables

**Table 1:** Sequence information of molecular markers used to screen 77 CAIGE barley lines.

Gene	Markers	Type	Forward Primer Sequence 5′-3′	Reverse Primer Sequence 5′-3′	Reference
*Rph3*	MLOC_198	CAPS	GCTGAGCCCCTAATACACGA	CCCATGTATGTGCTCGTTTG	Dinh et al., 2022 [16]
*Rph7*	Hvpg4	KASP-FAM3A1	GAAGGTGACCAAGTTCATGCTGTCATGCTCAAAACAGGTAACCCT		Chen et al., 2023 [17]
		KASP-HEX3A2	GAAGGTCGGAGTCAACGGATTGTCATGCTCAAAACAGGTAACCCA		
		KASP-R3C		GTATCCCTACGCAGTTATTTGACCTATG	
*Rph15*	K3	KASP-FAMA1	GAAGGTGACCAAGTTCATGCTGGGCTGTTATTAGCATGGTCCTC		Chen et al., 2021 [18]
		KASP-HEXA2	GAAGGTCGGAGTCAACGGATTGGGCTGTTATTAGCATGGTCCTG		
		KASP-K3RC		AATACCACAATGACTACCCCAGGTT	
*Rph20*	bPb-0837	DArT	GACACTTCGTGCCAGTTTG	CCTCCCTCCCTCTTCTCAAC	Hickey et al., 2011 [13]
*Rph20*	sun690-91	InDel	AACAAAAAGCGGCCGAAAAA	ACGGGCACATTGTGTCTATTT	Dracatos et al., 2021 [14]
*Rph23*	Ebmac0603	SSR	ACCGAAACTAAATGAACTACTTCG	TGCAAACTGTGCTATTAAGGG	Singh et al., 2015 [15]
*Rph24*	sun43-44	InDel	CTAGACACCACCACCACACC	ATACCAGAGTTTGCGTCCGG	Dracatos et al., 2021 [14]
*Rph28*	M5	CAPS	ATGATGTCACGGACGAGTCG	GCATCACCCTCC GTGTTGAT	Mehnaz et al., 2021 [11]

**Table 2:** Frequency and infection type response matrix of *Rph* genes postulated in CAIGE core panel (n = 77) using nine pathotypes.

Postulated Gene	No. of Lines	Pathotypes **								
		A	B	C	D	E	F	G	H	I
*Rph2*	8	R	R	S	R	R	S	S	S	S
*Rph3*	26	R	R	R	R	R	S	S	S	R
*Rph5*	1	R	S	R	R	R	R	R	R	R
*Rph9.am*	1	S	S	R	S	S	S	S	S	S
*Rph9.am+*	2	R	S	R	S	S	S	S	S	S
*Rph25*	3	S	R	S	S	S	S	S	S	S
*Rph25 + Rph9.am*	2	S	R	R	S	S	S	S	S	S
RphUASR *	3	R	R	R	R	R	R	R	R	R
Susceptible	22	S	S	S	S	S	S	S	S	S

* Nine additional lines carried UASR to at least one pathotype, which remained undetermined. ** A = 200 P-, B = 220 P+ *Rph13*+, C = 253 P+, D = 4610 P+, E = 5610 P+, F = 5457 P+, G = 5477 P-, H = 5656 P+, I = 5553 P+. R = resistant, S = susceptible.

**Table 3:** Identified all stage resistance (ASR) and adult plant resistance (APR) genes to barley leaf rust in an international barley panel (n = 77 genotypes).

Line ID	Origin	Genotype	ASR	APR ^a^	DS ^b^	APR Level ^c^
CG1	United States	Breeding line	*Rph2*	*Rph24*	8	Susceptible
CG2	Russia	Landrace	*UASR*	*Rph24*	3	HAPR
CG3	Armenia	Landrace	*Nil*	*Rph24*	7	LAPR
CG4	Pakistan	Landrace	*Nil*	*Rph24*	7.5	LAPR
CG5	Macedonia	Landrace	*Nil*		8.5	Susceptible
CG6	Spain	Landrace	*Rph9.am+*	*Rph24*	3	HAPR
CG7	Afghanistan	Landrace	*Nil*	*Rph24*	3	HAPR
CG8	United States	Landrace	*Nil*	*Rph24*	4.5	MAPR
CG9	China	Landrace	*Nil*	*Rph24*	7	LAPR
CG10	Macedonia	Landrace	*Nil*	*Rph24*	2.5	HAPR
CG11	Macedonia	Landrace	*Nil*	*Rph24*	3.5	HAPR
CG12	Russia	Landrace	*Nil*	*Rph24*	3	HAPR
CG13	Russia	Landrace	*Rph2*	*Rph24*	7.5	LAPR
CG14	Austria	Landrace	*UASR*	*Rph24*	6.5	LAPR
CG15	Azerbaijan	Landrace	*Nil*	*Rph24*	2.5	HAPR
CG16	Ethiopia	Single plant sel	*Nil*	*Rph24*	7	LAPR
CG17	Ethiopia	Single plant sel	*Nil*	*Rph24*	7	LAPR
CG18	Ethiopia	Single plant sel	*Nil*	*Rph24*	7	LAPR
CG19	Ethiopia	Single plant sel	*Nil*	*Rph24*	5	MAPR
CG20	Ethiopia	Single plant sel	*Nil*	*Rph24*	7	LAPR
CG21	Ethiopia	Single plant sel	*Nil*	*Rph24*	7.5	LAPR
CG22	Ethiopia	Single plant sel	*Nil*	*Rph24*	9	Susceptible
CG23	Greece	Single plant sel	*Rph9.am + Rph25*	*Rph24*	7	LAPR
CG24	Greece	Single plant sel	*UASR*	*Rph23*, *Rph24*	2	HAPR
CG25	Russia	Single plant sel	*Rph25*	*Rph24*	5	MAPR
CG26	Turkey	Single plant sel	*Rph2*	*Rph20*, *Rph24*	5	MAPR
CG27	Morocco	Breeding line	*Rph2*		8	Susceptible
CG28	Morocco	Breeding line	*UASR*	*Rph24*	7	*
CG29	Morocco	Breeding line	*UASR*		2.5	*
CG30	Syria	Breeding line	*Rph3*	*Rph24*	5	MAPR
CG31	Syria	Breeding line	*Rph3*		8	Susceptible
CG32	Morocco	Breeding line	*Rph3*		8	Susceptible
CG33	Morocco	Breeding line	*Rph3*	*Rph24*	5	MAPR
CG34	Morocco	Breeding line	*Rph3+*	*Rph20*, *Rph24*	3.5	*
CG35	Morocco	Breeding line	*Rph3*	*Rph23*	7.5	LAPR
CG36	Morocco	Genetic stock	*Rph3*	*Rph23*	7.5	LAPR
CG37	Morocco	Genetic stock	*Rph3*	*Rph23*	7.5	LAPR
CG38	Morocco	Genetic stock	*Rph3*	*Rph23*	7.5	LAPR
CG39	Morocco	Genetic stock	*Rph3*	*Rph23*	7	LAPR
CG40	Morocco	Genetic stock	*Rph3*	*Rph23*	7.5	LAPR
CG41	Morocco	Genetic stock	*Rph3*	*Rph23*	7.5	LAPR
CG42	Morocco	Unknown	*Rph3*		7	LAPR
CG43	Morocco	Breeding line	*UASR*	*Rph20*, *Rph24*	2	*
CG44	Morocco	Breeding line	*Nil*	*Rph20*, *Rph24*	3	HAPR
CG45	Morocco	Breeding line	*Rph25*	*Rph20, Rph24*	4	MAPR
CG46	Morocco	Breeding line	*Nil*	*Rph24*	7	LAPR
CG47	Morocco	Breeding line	*Rph3*		8	Susceptible
CG48	Morocco	Breeding line	*Rph3*	*Rph24*	7.5	LAPR
CG49	Morocco	Breeding line	*Rph3*		7.5	LAPR
CG50	Morocco	Breeding line	*Rph9.am*	*Rph23*	7.5	LAPR
CG51	Morocco	Breeding line	*Rph3*		8	Susceptible
CG52	Morocco	Breeding line	*Rph3*		8	Susceptible
CG53	Morocco	Unknown	*Rph9.am+*		7.5	LAPR
CG54	Morocco	Unknown	*Rph5*		3	*
CG55	Morocco	Genetic stock	*UASR*		3	*
CG56	Morocco	Unknown	*UASR*	*Rph23*	2	*
CG57	Morocco	Breeding line	*UASR*		2.5	*
CG58	Morocco	Unknown	*Rph3*		7	LAPR
CG59	Morocco	Breeding line	*Rph3*	*Rph23*	6	LAPR
CG60	Morocco	Advanced line	*Rph3*	*Rph23*	7	LAPR
CG61	Morocco	Breeding line	*Rph3*	*Rph23*	6	LAPR
CG62	Syria	Breeding line	*Rph2*	*Rph20*, *Rph24*	3	HAPR
CG63	Syria	Breeding line	*Nil*	*Rph20*, *Rph23*, *Rph24*	2.5	HAPR
CG64	Syria	Breeding line	*UASR*	*Rph20*, *Rph24*	2.5	*
CG65	Lebanon	Breeding line	*UASR*	*Rph20*, *Rph24*	4	MAPR
CG66	Lebanon	Breeding line	*Nil*	*Rph20*, *Rph24*	7	LAPR
CG67	Syria	Breeding line	*Rph3*	*Rph23*	6	LAPR
CG68	Lebanon	Breeding line	*Rph3*	*Rph23*, *Rph24*	4.5	MAPR
CG69	Lebanon	Breeding line	*Rph2*	*Rph24*	7	LAPR
CG70	Lebanon	Breeding line	*Rph3*		7.5	LAPR
CG71	Morocco	Breeding line	*Rph3*	*Rph24*	6	LAPR
CG72	Syria	Breeding line	*Rph2*	*Rph20*, *Rph24*	3	HAPR
CG73	Syria	Breeding line	*Rph25*	*Rph24*	3	HAPR
CG74	Syria	Breeding line	*UASR*	*Rph24*	6.5	LAPR
CG75	Syria	Breeding line	*Nil*	*Rph20*	5	MAPR
CG76	Syria	Breeding line	*Rph9.am + Rph25*	*Rph20*, *Rph23*, *Rph24*	2.5	HAPR
CG77	Syria	Breeding line	*Rph2* (segregating)	*Rph24*	7	LAPR

^a^ Assigned based on linked markers [(bPb-0837 and sun690-691 (*Rph20*); Ebmac0603 (*Rph23*); sun43-44 (*Rph24*)]. ^b^ DS = Disease score averaged over 2 replications on a 1–9 scale (assessed against field pathotype 5457P+). ^c^ APR levels determined as high (HAPR, DS = 1–3.5), moderate (MAPR, DS = 3.6–5), or low (LAPR, DS = 5.1–7.5). DS > 7.5 = susceptible. * Undetermined because of the presence of ASR to pt 5457P+ in these lines.

## Data Availability

Data are contained within the article and Supplementary Materials.

## Supplementary Materials

The following supporting information can be downloaded at: https://www.mdpi.com/article/10.3390/plants14203150/s1, Supplementary Table S1: Collection and passport data of 77 CAIGE lines tested in this study; Supplementary Table S2: Infection type (IT) response array produced by 12 selected *Puccinia hordei* pathotypes on a set of differential genotypes and genetic stocks representing 28 catalogued *Rph* genes; Supplementary Table S3: Infection type response array of 77 lines of CAIGE core panel using 9 pathotypes of *Puccinia hordei*.
